# Accuracy of a smart bottle in measuring fluid intake by American football players during pre-season training

**DOI:** 10.1038/s41598-023-38613-9

**Published:** 2023-07-14

**Authors:** Lindsay B. Baker, Shelby Alfred, Khalil A. Lee, Justina L. Bonsignore, Kristin L. Osterberg, Brandon Shepherd, Peter John D. De Chavez, Kobi Bentkovski

**Affiliations:** 1 Gatorade Sports Science Institute, PepsiCo R&D Life Sciences, 50 E. Stevens Ave., Valhalla, NY 10595 USA; 2 Gatorade Sports Science Institute, PepsiCo R&D Life Sciences, Bradenton, FL USA; 3 Data Science and Analytics, PepsiCo R&D, Plano, TX USA; 4 ImpacX.io Ltd, Rehovot, Israel

**Keywords:** Physiology, Urology

## Abstract

Rehydration is important for athlete performance and recovery. However, it can be challenging to follow appropriate fluid replacement practices due in part to difficulties in tracking fluid intake in real time. The purpose of this study was to determine the accuracy of a smart bottle in measuring fluid intake during exercise. Thirty male American football athletes drank from bottles equipped with a smart cap during outdoor pre-season practices (110 ± 30 min; 29.3 ± 3.0 °C; 75 ± 11% rh). The cap technology included optical sensors, microprocessors, batteries, and wireless connectivity that transmitted fluid volume data to a smartphone application in real-time. Reference measurements of fluid intake from the smart bottle were determined by gravimetry followed by conversion to volume using the density of the fluid consumed. There was no significant mean difference in fluid intake between the smart bottle and reference method (1220 ± 371 ml vs. 1236 ± 389 ml, p = 0.39 paired *t* test). Bland–Altman 95% limits of agreement between methods was − 212 to 180 ml. The smart bottle provided accurate measurements of fluid intake during exercise in real-world field conditions on a group level and within limits of agreement of − 212 to 180 ml (or approximately ± 15% of overall fluid intake) on an individual level.

## Introduction

Current consensus recommends that personalized hydration practices should be followed by athletes to attenuate the adverse effects of fluid-electrolyte imbalances on health and performance^1,2^. Several studies have assessed best practice methodologies and new technologies to conduct sweat testing to help inform athletes’ individualized fluid replacement needs^3,4^. However, the other important aspect of optimizing hydration status during activity is ensuring that fluid intake recommendations are followed by athletes. Observational studies have shown that some athletes accrue significant dehydration despite having access to fluid^5^, while others may overdrink relative to sweat losses during exercise^6,7^.

One of the challenges to following appropriate fluid replacement practices may be related to difficulties in knowing how much volume an athlete has ingested^8^. A possible approach to improve fluid intake tracking by athletes and/or their sports practitioners in real time is through the use of smart bottles, which have recently become commercially available. Previous studies have assessed various smart bottle technologies and reported their accuracy to be within 2–24% of reference measurements for 24-h fluid intake^9,10^. To date, most research with smart bottles has been related to their potential utility as a behavioral aid to improve hydration in recurrent kidney stone patients, as difficulty remembering to drink is a common barrier to sufficient fluid intake in this population^11–13^. By contrast, no studies have assessed the accuracy of a smart bottle technology when used by athletes in the context of real-world training conditions. If valid, smart bottle technology may also aid adherence to fluid intake recommendations in athletes.

American football players are among the more susceptible athletes to body fluid deficits, especially during summer pre-season training^14^; as the combination of warm environmental conditions and intense training can lead to substantial fluid loss through sweating^15–17^. For example, in one study with professional players of the National Football League (NFL) (124 ± 25 kg) sweating rate was 2.3 ± 0.4 L/h, which led to a 2.3 ± 1.1% body mass deficit over the course of a 2.2 h practice^18^. However, there is wide variation in sweat losses among studies, depending upon player size, environmental conditions, equipment worn, and training intensity, among other factors^19^. Normative data suggest that the range in sweating rate in American football is ~ 0.5 to 4 L/h^15^. Accordingly, a wide variation in fluid balance has also been observed in American football players^14^, with one study reporting a range of 0.0–2.8% change in body mass among NFL lineman and backs^20^.

Smart bottle technology may be a tool to help athletes and/or their sport practitioners better gauge fluid intake during exercise to prevent significant under- or overdrinking. However, the accuracy of smart bottle technology in measuring fluid intake during exercise in real-world field conditions is unknown. Therefore, the purpose of this study was to compare fluid intake measured with a smart bottle to standard reference fluid intake measurements during American football pre-season practice sessions.

## Methods

### Participants

Thirty male American football athletes participated in this study. All participants were members of the same post-graduate team based at the IMG Academy in Bradenton, Florida, USA. This study (clinical trial identifier: NCT04920266) was approved by the Sterling Institutional Review Board (sterlingirb.com) and has therefore been performed in accordance with the ethical standards in the Declaration of Helsinki. Each participant and his parent or guardian (for subjects under 18 years) were informed of the experimental procedures and associated risks before providing written informed consent or written informed assent (for subjects under 18 years).

### Study design

Testing took place over the course of three pre-season outdoor practices held in the early mornings during 1 week in August. The practices were led by team coaches and consisted of a range of activities including dynamic stretching, position-specific drills, and live scrimmaging. The intensity and duration (75–130 min) of the practices, as well as the breaks in activity were not influenced by the investigators. Players wore helmets, shoulder pads, jersey top, shorts, and cleats during practices. The temperature and relative humidity during practice were measured with a Kestrel 5400 (Nielsen-Kellerman Co.) positioned on the sideline. Each of the 30 players participated in the study during one of the three practices (i.e. n = 10 per practice). One player did not practice because of injury but still participated in the study by drinking ad libitum on the sideline.

### Measurements

During practice, players drank ad libitum from 0.9 L-squeeze bottles equipped with a smart cap (Gatorade Smart G^x^ Bottle, described in more detail below). The athletes were allowed to drink during substitutions as well as scheduled drink breaks for a total of ~ 3–6 opportunities to drink per practice. Reference measurements of fluid intake from the smart bottle were determined by gravimetry (change in bottle mass) using a compact digital bench scale (Ohaus CS2000, with a 1 g accuracy) followed by conversion to volume using the density of the fluid consumed (6% carbohydrate-electrolyte solution). Bottle measurements using both the smart bottle technology and the reference method were obtained before practice, before/after refills during practice (as needed), and after practice to determine total fluid intake for each participant. The bottles sat resting on a flat surface (i.e. table) during the measurements. Fluid volume measurements were conducted in triplicate at each time point to assess reliability. The first value of the triplicate measurements at each time point was used in the calculation for fluid intake. Bottles were stored in ice chests on the sideline and players drank from their individually labeled bottles during breaks in practice.

Before and after practice, body mass was measured to the nearest 0.01 kg (Mettler Toledo, Model PBD655-BC300). Players wore compression shorts only and were asked to towel dry prior to stepping on the scale. Post-exercise fluid balance was calculated as the difference between pre- and post-practice body mass. Whole body sweating rate was calculated as the difference between pre- and post-practice body mass adjusted for fluid consumed (based on reference measurement of fluid intake). No food was consumed and there was no urine/stool loss between pre- and post-practice body mass measurements. Three players did not weigh in before practice and one player did not weigh out after practice. Therefore, the sample size was n = 26 for fluid balance and WBSR data. All data were recorded on paper by study investigators. After the training sessions, the lead author entered data into a digital spreadsheet and a coauthor checked the accuracy of the inputs.

### Smart bottle

The Smart Bottle used in this study was a 0.9 L plastic squeeze bottle with technology integrated into the cap—including sensors, microprocessors, batteries, and wireless connectivity—that detects the level of liquid inside the bottle and transfers the fluid volume data to a smartphone application. The sensors in the cap measure the liquid level inside the bottle via a laser optical method based on the Time of Flight (ToF) principle. ToF is the time it takes emitted photons to travel from the cap, through the air gap below the cap, and bounce back (reflect) from the level of the liquid (the target). The ranging distance is calculated by the time difference between the emission and the reception of the photons, providing the actual distance of the target in millimeters. The distance between the cap and the top of the liquid is then correlated to volume in milliliters based on the bottle’s geometry. The Smart Bottle needs to be resting on a flat surface within 10 m of the smartphone to make fluid volume measurements and the data are transferred to the application 30–60 s after measurement initiation. To collect fluid volume data from multiple bottles a separate smartphone application/device needs to be paired to each bottle.

### Statistical analysis

Analyses were carried out using XLSTAT (version 2021.4.1; Addinsoft), Minitab 19 Statistical Software (Minitab Inc.), and NCSS 12 Statistical Software (NCSS, LLC). Values are presented as mean ± SD. Scatterplots and Pearson correlation coefficients were obtained to assess the relation between the smart bottle and reference methods for fluid intake. Paired *t* tests were used to evaluate the difference in fluid intake between methods. Bland–Altman plots for predicted versus measured fluid intake were used to illustrate results by individual participants and 95% limits of agreement (LOA) were set around the mean difference (line of bias). A correlation calculation between the outcomes of the y-axis (difference between smart bottle and reference method) and x-axis (average of smart bottle and reference method) of the Bland–Altman plot was conducted to determine if there was a significant difference in over or under-estimations when taking into account the total volume consumed (low vs high drinkers). An orthogonal (Deming) regression was conducted, whereby the slope and intercept of the regression line and line of identity of the scatterplot were compared, to determine the accuracy of the smart bottle in measuring fluid intake. Shapiro–Wilk tests were used to assess the normality of the residuals. For practical context, fluid intake prediction error was also expressed as a percentage of body mass for each player. Pre-practice body mass was used for this calculation except for n = 3 players who did not weigh in before practice, in which case their post-practice body mass was used. The significance level for all statistical tests was set at alpha = 0.05.

## Results

The participants age and body mass were 18 ± 1 years and 92.4 ± 20.4 kg, respectively. Practice duration was 110 ± 30 min and the conditions on the field were 29.3 ± 3.0 °C and 75 ± 11% relative humidity. The players drank 1236 ± 389 ml of fluid during practice and their whole body sweat loss was 1.95 ± 0.82 L. Overall fluid balance at the end of practice was − 0.64 ± 0.73%. A breakdown of the descriptive data by practice day are shown in Table 1.

**Table 1:** Descriptive data across the three practice days.

	Practice 1	Practice 2	Practice 3
N	10	10	10
Participant age (years)	18 ± 1	18 ± 1	18 ± 1
Body mass (kg)	90.6 ± 20.7	93.9 ± 23.5	92.1 ± 21.2
Exercise duration (min)	130	125	75
Air temperature (°C)	30.3 ± 3.5	30.0 ± 2.1	26.9 ± 1.9
Relative humidity (%)	70.6 ± 12.8	72.9 ± 6.6	86.8 ± 4.8
Fluid balance (% change in body mass from pre-exercise)	− 1.17 ± 0.60	− 0.55 ± 0.70*	− 0.09 ± 0.45^#^
Reference method fluid intake (ml)	1419 ± 422	1301 ± 269	989 ± 357
Smart bottle fluid intake (ml)	1387 ± 390 (p = 0.24)	1273 ± 248 (p = 0.55)	1001 ± 376 (p = 0.55)

Values are mean ± SD. P values are for paired *t* test comparing smart bottle vs. reference method fluid intake.

*n = 9, ^#^n = 7.

When using the smart cap to measure bottle fluid volume the mean coefficient of variation (CV) across all 110 measurement time points (i.e. when full, partially full, and empty) was 18%. The absolute variation among the triplicate measurements was 38 ± 40 ml (range: 0–234 ml) with the smart cap. The mean CV using the Ohaus scale to measure fluid mass was 0.1% and varied by no more than ± 2 g among triplicate measurements.

**Figure 1:**
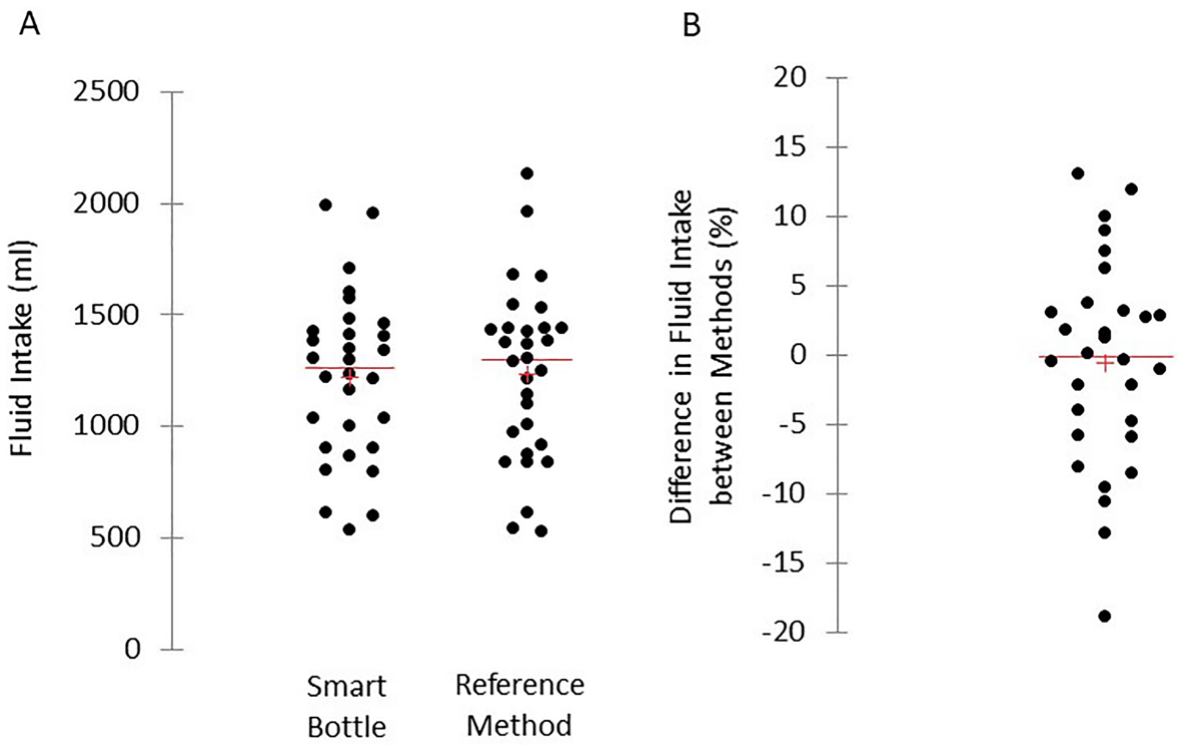
(**A**) Scattergram showing data for fluid intake measured with the smart bottle and reference method (n = 30). There was no significant mean difference in total fluid intake between methods (p = 0.39 paired *t* test). (**B**) Scattergram showing difference in fluid intake between methods expressed as a percentage (n = 30). Horizontal line represents the group median, and the plus sign represents the mean.

**Figure 2:**
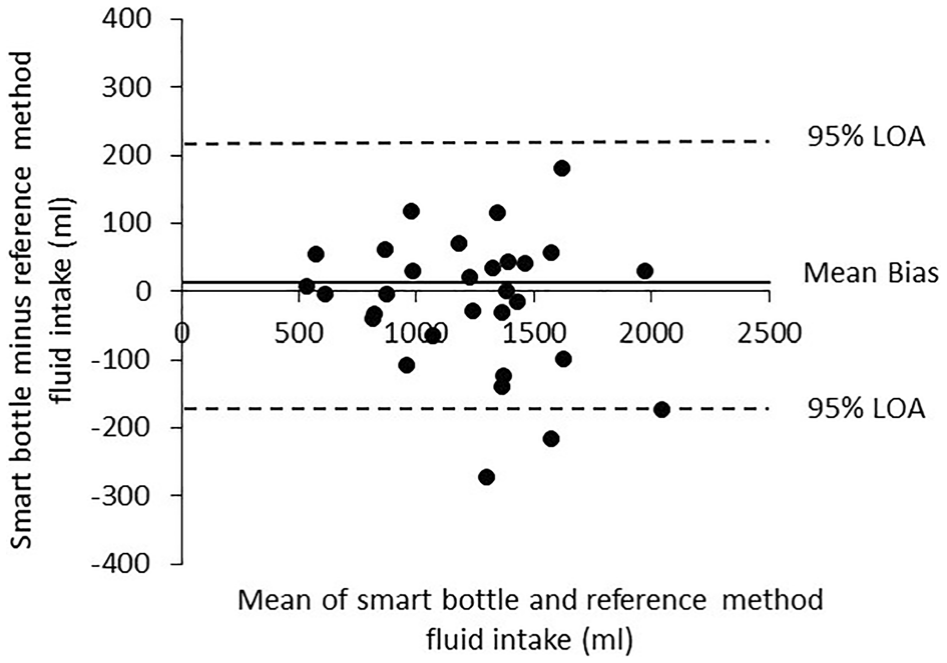
Bland–Altman plot comparing fluid intake values measured using the reference method versus fluid intake values measured with the smart bottle (n = 30). The mean bias between methods was 16 ml. The lower and upper bounds of the 95% Limits of Agreement (LOA) were − 212 and 180 ml, respectively.

The mean and individual data for fluid intake measured with the smart bottle and reference method are shown in Fig. 1A. There was no significant mean difference in total fluid intake between the smart bottle and reference method (1220 ± 371 ml vs. 1236 ± 389 ml, p = 0.39 paired *t* test). The mean difference between methods expressed as a percentage was − 0.6 ± 7.5%. The 95% confidence interval (CI) for the percentage mean difference was − 3.4% (lower bound) to 2.2% (upper bound) (Fig. 1B). Figure 2 shows the Bland–Altman plot comparing fluid intake values measured using the reference method versus fluid intake values measured with the smart bottle. The mean bias between methods was 16 ml. The lower and upper bounds of the 95% LOA were − 212 and 180 ml, respectively. There was no correlation between the outcomes of the y-axis (difference between smart bottle and reference method) and x-axis (average of smart bottle and reference method) of the Bland–Altman (r = − 0.18, p = 0.33).

**Figure 3:**
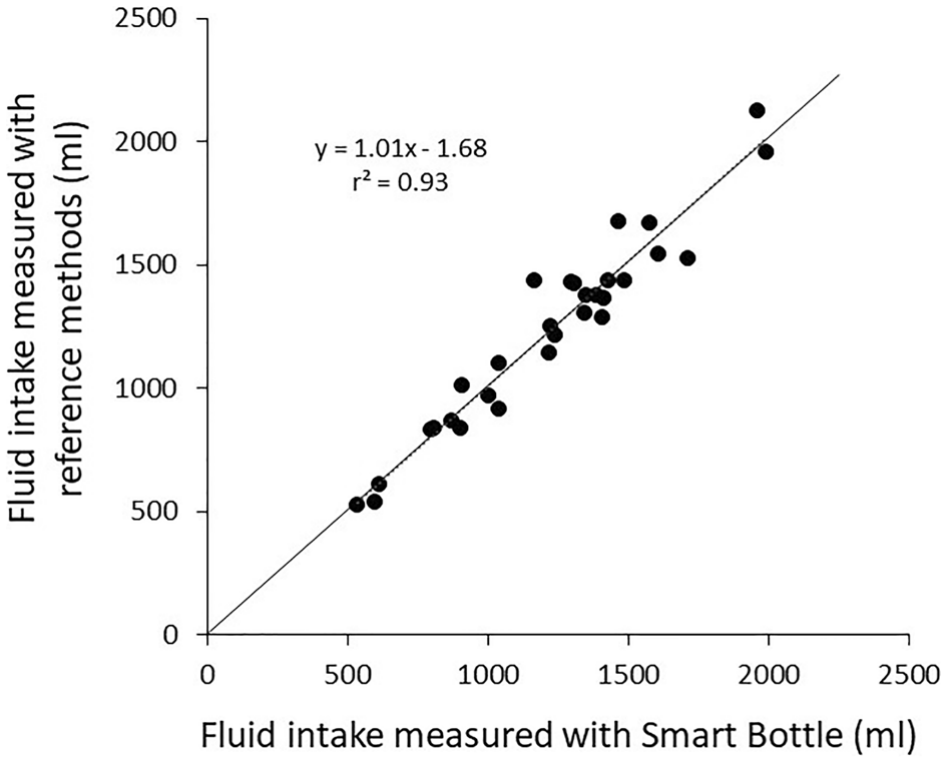
Scatterplot of smart bottle versus reference fluid intake (n = 30; r = 0.96, p < 0.0001). The slope and intercept of the regression line were not different from one (95% confidence interval: 0.95, 1.15) and zero (95% confidence interval: − 178, 85), respectively.

As shown in the scatterplot in Fig. 3, there was a significant correlation between the smart bottle and reference methods of fluid intake (r = 0.96, p < 0.0001). Based on the Deming regression analysis, the slope and intercept of the regression line describing the smart bottle vs. reference method of fluid intake were not different than one (95% CI 0.95, 1.15) and zero (95% CI − 178, 85), respectively.

**Figure 4:**
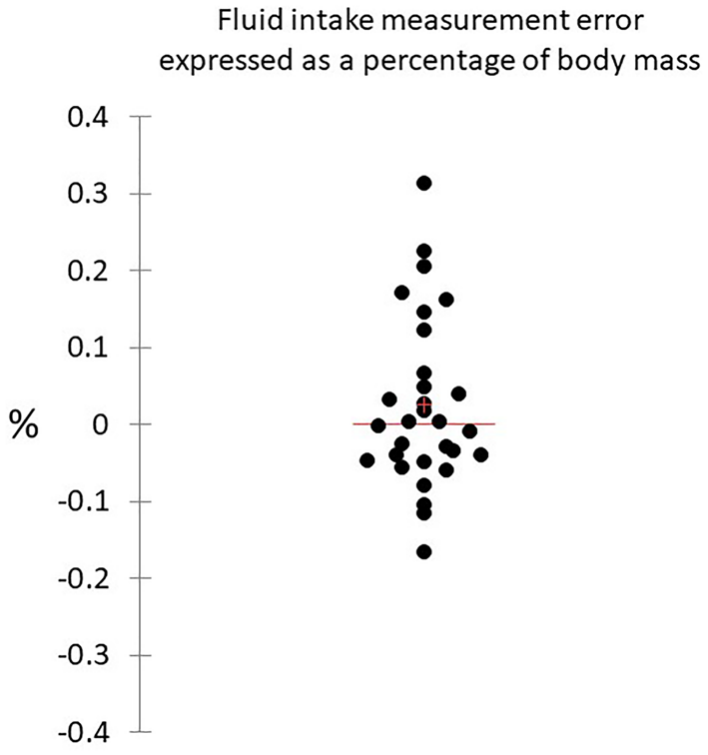
Scattergram showing the fluid intake measurement error expressed as a percentage of body mass for each participant (n = 30). Horizontal line represents the group median, and the plus sign represents the mean.

For practical context the fluid intake measurement error of the smart bottle was expressed as a percentage of the athlete’s body mass (Fig. 4). Based on these results fluid balance would be altered by + 0.31% to − 0.17% during practice due to the difference between fluid volume estimated by the smart bottle and actual fluid intake volume.

## Discussion

This study determined the accuracy of a smart bottle in measuring ad libitum fluid intake by American football athletes during pre-season outdoor training sessions. The smart bottle used in this study was a squeeze bottle with optical sensors integrated into the cap that detect the level of liquid inside the bottle and Bluetooth connectivity to transfer the fluid volume data to a smartphone application. The main finding was that there was no significant mean difference in total fluid intake between the smart bottle and reference method. Further, the slope and intercept of the regression line describing the smart bottle versus the reference method were not different from one and zero, respectively. Bland–Altman 95% Limits of Agreement between methods was − 212 to 180 ml, which translates to approximately ± 15% of the players’ overall fluid intake. Therefore, the smart bottle is a valid and practical tool that provides fluid intake measurements within ± 15% of standard reference methods during exercise in real-world field conditions. When expressed as a percentage of the athletes’ body mass, this measurement error translates to a 0.31% overestimation to 0.17% underestimation in overall fluid balance. When combined with sweat testing to help inform athletes’ individualized fluid replacement needs, a smart bottle could help close the loop on real-time hydration. By having access to the fluid volume data through the smartphone application, the individual athlete or their sports practitioner could monitor fluid intake and compare it against pre-determined fluid intake goals that were based on sweat loss^21^.

Previous studies have assessed various smart bottle technologies, including capacitive sensors extending from the bottle cap or weight sensors mounted on the bottle base. Cohen et al.^10^ tested the accuracy of four commercially available smart bottles (n = 1 each) during a controlled phase of 50 sips (known volumes poured out by hand) and a free-living phase of 50 sips (actual drinking events). The cumulative mean percentage error compared with ground truth weight-based measurements after 100 total sips ranged from ~ 2 to 24% across the four bottles tested. The capacitive sensor-based bottles had a lower mean error (1.9% and 2.1%) than the weight sensor-based bottles (− 13.3% and − 24.1%)^10^. In another study^9^, the accuracy of a smart bottle with a capacitive touch sensing technology was tested over a two-week period in a free-living setting with eight participants. Daily smart bottle measurements of fluid volume were compared against the participants’ measurements made by hand using a measuring cup. The overall mean difference between hand and smart bottle measurements was 0.0%, with a 95% confidence interval of − 3% to 3%^9^. Similarly, the mean fluid volume error in the present study was − 0.6 ± 7.5% for the 30 smart bottles tested, with a 95% confidence interval of − 3.4% to 2.2%.

Questionnaires have also been used to estimate fluid intake during exercise. For example, Wardenaar et al.^22^ compared observed fluid intake versus estimated fluid intake via the Food and Fluid Exercise Questionnaire (FFEQ) in 58 recreational endurance athletes competing in four different running events and one cross duathlon in the Netherlands. The FFEQ included questions on food and fluid intake during exercise as well as photographs for different serving sizes of beverages to facilitate the reporting of fluids. In this study the median observed fluid intake was 262 ml/h compared with 268 ml/h estimated from the FFEQ. Based on the Bland–Altman analysis there was a non-significant mean bias of 18 ± 106 ml/h and 95% LOA of − 198 to 225 ml/h^22^, equating approximately to a possible − 76% to 86% measurement error on an individual level. By comparison, in the present study the mean reference method fluid intake was 1236 ± 389 ml compared with 1220 ± 371 ml estimated from the smart bottle, with a 95% LOA of − 198 to 225 ml (equating to − 17% to 15% of overall fluid intake). Thus, while a questionnaire may accurately estimate fluid intake on a group level during competitions in recreational athletes, there seems to be potential for larger measurement error on an individual level compared with smart bottle technologies. However, a study using all three methods of assessing fluid intake (questionnaire, smart bottle, and reference) would be needed to make direct comparisons.

This study builds upon previous literature by reporting the accuracy of a novel optical sensor-based smart bottle when used by athletes in the context of real-world training conditions. The testing took place in ecologically valid conditions, whereby athletes were able to drink ad libitum and use the smart bottles at free-will during breaks in practice. In addition, the bottles experienced large temperature variations, from storage in ice chests during transport and on the sideline to the warm/humid outside air when being used by athletes. Furthermore, for practical interpretation of results the fluid intake measurement error of the smart bottle was expressed as a percentage of athlete body mass. Based on these results overall fluid balance would be altered by + 0.31% to − 0.17% during practice due to the difference between smart bottle and actual fluid intake volumes, which is likely of practical and clinical insignificance.

In agreement with previous observations^15^, sweating rates (0.47–2.51 L/h) and total sweat losses (0.77–3.72 L) varied considerably among American football players, with some experiencing significant fluid losses as a result of the high intensity practice and warm/humid conditions. Fluid balance also varied substantially among athletes; ranging from 2.35% loss to 0.47% gain during the practice sessions, which is similar to previous observations in American football^14,20^.

A limitation of the present study was that each player was tested only once to make within subject comparisons between the smart bottle and reference method for measuring fluid intake. It would be useful in future research to assess within person (day-to-day) variation of the measurement as well as compare ad libitum fluid intake behavior between the smart bottle and standard squeeze bottle. Previous research has reported that everyday use of a smart bottle improved hydration (24-h urine volume) in stone formers by helping them remember to drink water^11^. Whereas another study found the use of a smart water bottle did not further augment urine output in kidney stone patients beyond counseling alone^13^. Future research is needed to determine the utility of smart bottle technology in improving hydration practices and fluid balance during physical activity or sports practices/competition. In addition, future research is needed to determine the accuracy of the smart bottle at various beverage temperatures (e.g. ranging from warm to cold).

In summary, this study tested the accuracy of a smart squeeze bottle that measures fluid intake in real time via cap-integrated optical sensors. There was no significant mean difference in total fluid intake between the smart bottle and reference method when tested with American football players during pre-season training sessions. Bland–Altman 95% limits of agreement between methods was − 212 to 180 ml. Therefore, the smart bottle provided accurate measurements of fluid intake during exercise in real-world field conditions on a group level and within limits of agreement of approximately ± 15% of overall fluid intake on an individual level. This technology represents a valid and practical tool to measure fluid intake in real-time, which may help facilitate adherence to personalized hydration strategies during exercise.

## Data Availability

The datasets generated during and/or analyzed during the current study are available from the corresponding author on reasonable request.
